# HEV-ORF3 Encoding Phosphoprotein Interacts With Hepsin

**DOI:** 10.5812/hepatmon.13902

**Published:** 2014-01-18

**Authors:** Chunyan Wang, Liang Guo, Dayi Yu, Xiuguo Hua, Zhibiao Yang, Congli Yuan, Li Cui

**Affiliations:** 1 Shanghai Key Laboratory of Veterinary Biotechnology, School of Agriculture and Biology, Shanghai Jiao Tong University, Shanghai, China; 2 Animal Disease Control Center of Min Hang District, Shanghai, China

**Keywords:** ORF3 protein, Hepatitis E virus, Hepsin, Two-Hybrid System Techniques, Immunoprecipitation

## Abstract

**Background:**

Hepatitis E virus (HEV) is a major causative agent of acute clinical hepatitis in adults through much of Asia, the Middle East and Africa. Open reading frame 3 (ORF3) encodes around 120 amino acids of phosphorylation protein that associates with the cytoskeleton, while its precise biological function is still unknown.

**Objectives:**

In order to understand the function of ORF3 protein (pORF3) in depth, HEV ORF3 interacting proteins were screened in human hepatocytes cDNA library using two-hybrid system techniques and further verification of the interactions were carried out through co-immunoprecipitation (Co-IP).

**Materials and Methods:**

The Cyto-Trap two-hybrid system technology, a classical method for analyzing protein interactions, was used to screen the pORF3 interacting proteins from human hepatocytes cDNA library.

**Results:**

Through the Cyto-Trap two-hybrid system, eight proteins interacting with pORF3 were winnowed. The Co-IP results confirmed that hepsin which is reported to function as the inhibitor of several tumors reacted with pORF3.

**Conclusions:**

Out of eight screened proteins interacting with pORF3, hepsin was confirmed to have specific interactions with pORF3.

## 1. Background

Hepatitis E virus (HEV), a member of the family Hepeviridae, is believed to be a major pathogen responsible for endemic infections as well as large epidemics of acute clinical hepatitis (1). Infection caused by Hepatitis E virus (HEV) has been a significant global health concern and has been identified and described as a zoonotic infection by earlier researchers (2). Epidemics of HEV infection has been reported in many developing countries of Asia and Africa, however specific sporadic cases of HEV infection have also been reported in industrialized countries (3). Among the known subtypes, genotype 4 hepatitis E virus has been a predominant pathogen infringing the Chinese, especially since the last decade or so (4, 5). The genome of HEV is a single-strand RNA of 7.2 kbs that is positive-sense with 5’-methylgunine cap and 3’ ploy (A) stretch and contains three partially overlapping open reading frames (ORF), called ORF1, ORF2 and ORF3 (6, 7). ORF3 encodes around 120 amino acids of phosphoprotein that can associate with the cytoskeleton using one of its hydrophobic domains and can homodimerize through a 43-amino-acid interaction domain (6). Previous researches have suggested that the pORF3 can promote cell survival and proliferation and dampen innate host response through an attenuated acute phase response (8). Meanwhile, the pORF3 is important to virion egress from infected cells and the PSAP motif has a justified role as a functional domain for HEV budding (9, 10).

## 2. Objectives

The major objectives of the current study were to screen HEV-pORF3 interacting proteins from hepatocytes cDNA library using yeast two-hybrid systems, and to further confirm the interactions by Co-IP and western-blotting assays. These novel research findings will hopefully bring new descriptions to better understand the specific function of pORF3.

## 3. Materials and Methods

### 3.1. Materials

Preservation of genotype 4 hepatitis E virus was carried out at our laboratory. The Cyto-Trap two-hybrid system used in the current study including pMyr and pSos vectors, CDC25H cells and corresponding control plasmids were purchased from Stratagene. The pcDNA3.1-His-Flag-tag (pcDNA3.1 HF), a mammalian expression vector derived from pcDNA3.1 was purchased from Life Technologies and modifications for purification were carried out through His-tag and Co-IP with Flag-tag. The vector M51 (Genecopoeia, America) was used to explore the coordinated co-expression of two genes with the same vector and the Green fluorescent protein (GFP) sequence, which allowed for direct observation of expression results.

### 3.2. Plasmid Construction

To construct the pSos-bait ORF3 for Cyto-Trap two-hybrid system assay, the ORF3 cDNA of HEV was amplified using the primers listed in Table 1 and cloned into pSos vector by BamH I and Sal I restriction sites. To generate an N-terminal with HF-tagged ORF3 expression construct for Co-IP, the cDNA of ORF3 was amplified using the primers listed in Table 1 and the PCR product was cloned into the pcDNA3.1-His-Flag vector via the Hind III and EcoR I restriction sites to create pcDNA3.1-HF-ORF3. The gene of hepsin (HPN) of a transmembrane serine protease corresponding to bases from 132 bp to 1250 bp was amplified through primers presented in Table 1 and the resulting amplification product was then sub-cloned into the M51 vector for Co-IP.

**Table 1.** Primers Applied in This Research

Target	Primers Sequence (5’-3’)	
	Forward	Reverse
**pSos-bait-ORF3**	caggatccggatccgatgccacc	cagtcgacacgcgttcaacggcgaag
**pcDNA3.1-HF-ORF3**	atcgcagccaagcttatggagat-accaccatccatgcgct	attcgcccggaattctc - aacggcgccccag
**M51-HPN **	agaccaccaagcttaggcgcaaggagggt	agtcgaccgaattctcagagctgggtcaccatgcc

### 3.3. Screening of Interacting Proteins by Yeast Two-Hybrid System

During this study, cytoplasm-based yeast two-hybrid systems were adopted, to dissect the proteins interacting with pORF3 from human hepatocytes cDNA library. All experiments were carried out strictly according to the manufacturer’s instructions. Briefly, the plasmids of pSos-ORF3 as baits and that of human hepatcytes cDNA library were co-transformed into CDC25H cells maintained in the absence of leucine and uracil in plates containing glucose but not galactose at 25°C. Co-transformation of CDC25H was performed with pSos/pMyr functioning as a negative control, whereas pSos-MafB/pMyr-MafB was regarded as a positive control. However, screening of putative clones was carried out on the basis of their ability to grow on minimal synthetic medium in short of leucine and uracil on plates containing galatose but not glucose at 37°C. Further, the assumed positive clones were re-transformed into the same CDC25H cells with pSos to confirm the interactions. The extracted positive plasmids were sent for sequencing by the recommended primers Cyto-Trap two-hybrid system and the sequencing outcomes were then blasted in GenBank.

### 3.4. Co-Immunoprecipitation (Co-IP)

In this study, the recombinant pcDNA3.1HF-ORF3 and M51-HPN were transformed into 293 T cells according to the Lipofectaminate 2000 (Life Technologies) instructions. After 48 hours of expression, cells were washed twice with PBS and lysed in a buffer containing 50 mM Tris-HCL pH 7.4, 150 mM NaCl and 1% Triton X-100 and added 2 µL phenylmethylsulfonyl fluoride (PMSF) for 30 minutes at 4°C on a rotator. The anti-Flag M2 affinity gel was prepared in advance and the unbound cell antibody against flag was washed away by TBS buffer (150 mM Tris HCL with 150 mM NaCl, pH 7.4). 1 mL of total protein lysates were incubated in 20 µL of beads, overnight at 4°C on a shaker. Beads were then washed 3 times in TBS buffer and precipitates were resolved on an 8% gel by SDS-PAGE and electro-transferred to immoblin-P membrane (Merck Millipore) in 50 mM Tris-boric acid at 4°C for 2 hours at 300 mA. Membranes were blocked with 5% non-fat milk powder in TBS-Tween or TBST (50 mM Tris-HCL pH 8.0, 150 mM NaCl, 0.4% Tween-20) for 1 hour at room temperature. The anti-Flag were diluted in the TBST buffer and incubation of the membrane on shaker was carried out overnight at 4°C, and then anti-mouse was added to 10 mL of TBST and incubated for 1 hour at room temperature. Finally, membranes were washed in TBST and detected by chemluminescence. 

## 4. Results

From the above mentioned experiments on interaction between pORF3 and hepsin, the following results were recorded.

### 4.1. Identification of Interacting Proteins

In order to identify the interacting proteins, the ORF3 was inserted into the pSos vector to construct the pSos-ORF3 recombinant plasmid. The results of sequencing and western blotting techniques exploited that the bait plasmid can be used for subsequent library screening. The pSos-ORF3 and the human hepatocyte cDNA library plasmids were co-transformed to the temperature-sensitive yeast strain cdc25H cells. Transformed yeast cdc25H cells with specific galactose-induced growth at the per-missive temperature (37°C) were selected. 15 putatively positive clones were screened and co-transformed to the same cells once again with pSos for further verification. After re-verification, eight positive clones were confirmed and their cDNAs were further analyzed by means of DNA-sequencing and identified using the GenBank Blastp program (Table 2). The hepsin, a transmembrane protease, was selected to verify the interaction with the pORF3 through Co-IP. 

**Table 2.** The Blast Results of Positive Clone Within in GenBank

Clone	Name and Location	Homology, %
**821**	Homo sapiens albumin (ALB), bases: 72 to 1151	96
**475**	Homo sapiens mitochondrion genome, bases: 15065 to 15889	99
**763**	Homo sapiens chromosome 3 genomic contig, bases: 36379 to 36479	96
**194**	Homo sapiens histidine-rich glycol-protein (HRG), bases: 823 to 1679	99
**255**	Transmembrane protease, serine1 (HPN), bases:132 to 1250	94
**376**	Homo sapiens phosphorinositide-3-kinase (p110δ), bases: 3589 to 4661	95
**788**	Complement component 3, bases: 2988 to 3998	97
**72**	Homo sapiens ribosomal protein S11 (RPS11), bases: 16 to 613	98

### 4.2. Co-Immunoprecipitation (Co-IP)

**Figure 1.**
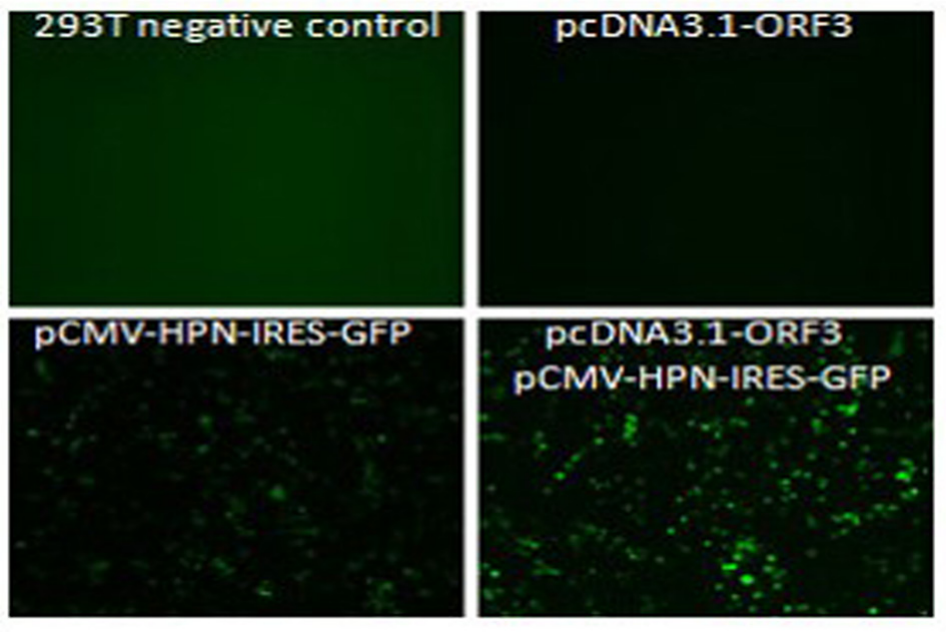
Fluorescence Microscopy Detection Result for Plasmid Transfection Shows the Success of Transformation of Recombinant Plasmids

**Figure 2.**
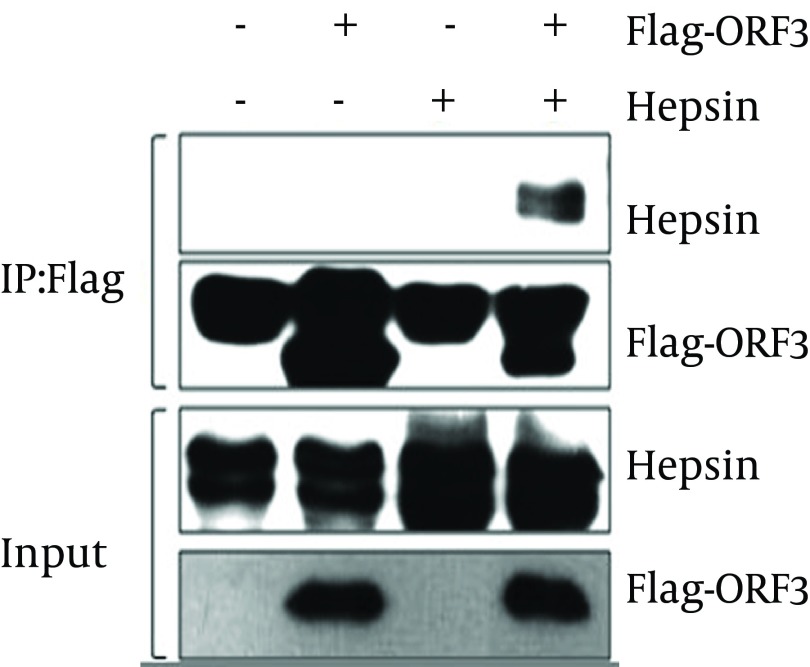
The Western Blotting Result of Co-IP Indicates The Interaction Between pORF3 and Hepsin

For verification of the interaction established by the yeast two-hybrid screening, we used a Co-IP assay. With the Green fluorescent protein (GFP) sequence in M51 vector, the fluorescence microscopy detection results of M51-HPN (pCMV-HPN-IRES-GFP) presented green fluorescence, which reflects the success of transformation and construction of recombinant plasmid (Figure 1). Our Co-IP and western blotting results indicated that HPN interacts with pORF3, specifically pORF3 in human cells (Figure 2).

## 5. Discussion

The current study was carried out to investigate the interactions between HEV-pORF3 and hepsin. Hepatitis E virus (HEV) is the causative agent of hepatitis E, a form of acute viral hepatitis that is endemic in developing countries. It is estimated that about 2 billion, a third of the world’s population, live in areas where they are at risk of HEV infection (3, 4). The pORF3 is definitely an essential element for the establishment of HEV infections in animals but its exact functions are still unclear and obscure. Previous studies have suggested that pORF3 prolongs endomembrane growth factor signaling and also promotes cell survival to contribute positively in viral replication and pathogenesis, and PASP motif in pORF3 is responsible for viron egress from infected cells (8-11).

In the present study, yeast two-hybrid system was employed to select the pORF3 interacting proteins from human hepatacytes cDNA library. Eight selected positive clones and their DNA sequences were analyzed through DNA sequencing and the GenBank Blastp program (Table 2) was used for identification. Hepsin, a type II transmembrane serine protease, was selected for further studies and confirmation of outcomes. Results of Co-IP and western blotting techniques indicated that the hepsin has specific interactions with pORF3.

It has been reported that hepsin, a cell surface protease is associated with growth and progression of cancers, particularly prostate cancer and its over-expression is found in more than 90% of human prostate cancer cases (12-14). Hepsin is also thought to be involved in diverse cellular functions, including blood coagulation and the maintenance of cell morphology (15, 16). Furthermore, the ORF3 protein is likely to cause an imbalance in physiological processes like coagulation and fibrinolysis by interacting with certain host proteins and eventually triggering the corresponding pathological processes (17). Therefore, from novel findings of the current study, it was concluded that pORF3-Hepsin interactions might lead to certain disturbances and imbalances in coagulation; we suggest further detailed and specific studies to unfold the domain of interaction and the real biological significance of pORF3-Hapsin interactions.

## References

[1] Emerson SU, Purcell RH (2003). Hepatitis E virus.. Rev Med Virol..

[2] Vasickova P, Psikal I, Kralik P, Widen F, Hubalek Z, Pavlik I (2007). Hepatitis E virus: a review.. Veterinarni Medicina..

[3] Chandra V, Taneja S, Kalia M, Jameel S (2008). Molecular biology and pathogenesis of hepatitis E virus.. J Biosci..

[4] Shen Q, Ren R, Zhang W, Yang Z, Yang S, Chen Y (2011). Prevalence of hepatitis E virus and porcine caliciviruses in pig farms of Guizhou province, China.. Hepat Mon..

[5] Wang H, He Y, Shen Q, Wang X, Yang S, Cui L (2012). Complete genome sequence of the genotype 4 hepatitis E virus strain prevalent in swine in Jiangsu Province, China, reveals a close relationship with that from the human population in this area.. J Virol..

[6] Ahmad I, Holla RP, Jameel S (2011). Molecular virology of hepatitis E virus.. Virus Res..

[7] Meng XJ (2010). Recent advances in Hepatitis E virus.. J Viral Hepat..

[8] Yamada K, Takahashi M, Hoshino Y, Takahashi H, Ichiyama K, Nagashima S (2009). ORF3 protein of hepatitis E virus is essential for virion release from infected cells.. J Gen Virol..

[9] Kenney SP, Pudupakam RS, Huang YW, Pierson FW, LeRoith T, Meng XJ (2012). The PSAP motif within the ORF3 protein of an avian strain of the hepatitis E virus is not critical for viral infectivity in vivo but plays a role in virus release.. J Virol..

[10] Nagashima S, Takahashi M, Jirintai, Tanaka T, Yamada K, Nishizawa T (2011). A PSAP motif in the ORF3 protein of hepatitis E virus is necessary for virion release from infected cells.. J Gen Virol..

[11] Chandra V, Kar-Roy A, Kumari S, Mayor S, Jameel S (2008). The hepatitis E virus ORF3 protein modulates epidermal growth factor receptor trafficking, STAT3 translocation, and the acute-phase response.. J Virol..

[12] Chen M, Chen LM, Lin CY, Chai KX (2010). Hepsin activates prostasin and cleaves the extracellular domain of the epidermal growth factor receptor.. Mol Cell Biochem..

[13] Holt SK, Kwon EM, Lin DW, Ostrander EA, Stanford JL (2010). Association of hepsin gene variants with prostate cancer risk and prognosis.. Prostate..

[14] Nandana S, Ellwood-Yen K, Sawyers C, Wills M, Weidow B, Case T (2010). Hepsin cooperates with MYC in the progression of adenocarcinoma in a prostate cancer mouse model.. Prostate..

[15] Hsu YC, Huang HP, Yu IS, Su KY, Lin SR, Lin WC (2012). Serine protease hepsin regulates hepatocyte size and hemodynamic retention of tumor cells by hepatocyte growth factor signaling in mice.. Hepatology..

[16] Sampson MT, Kakkar AK (2002). Coagulation proteases and human cancer.. Biochem Soc Trans..

[17] Zhang W, Yang S, Ren L, Shen Q, Cui L, Fan K (2009). Hepatitis E virus infection in central China reveals no evidence of cross-species transmission between human and swine in this area.. PLoS One..

